# A Deep Regression Model for Tongue Image Color Correction Based on CNN

**DOI:** 10.3390/jimaging11110381

**Published:** 2025-10-29

**Authors:** Xiyuan Cao, Delong Zhang, Chunyang Jin, Wei Zhang, Zhidong Zhang, Chenyang Xue

**Affiliations:** 1 State Key Laboratory of Extreme Environment Optoelectronic Dynamic Testing Technology and Instrument, North University of China, Taiyuan 030051, China; 2 School of Instrument and Electronics, North University of China, Taiyuan 030051, China

**Keywords:** color correction, CNNs, deep learning, tongue features

## Abstract

Different viewing or shooting situations can affect color authenticity and generally lead to visual inconsistencies for the same images. At present, deep learning has gained popularity and opened up new avenues for image processing and optimization. In this paper, we propose a novel regression model named TococoNet (Tongue Color Correction Network) that extends from CNN (convolutional neural network) to eliminate the color bias in tongue images. The TococoNet model consists of symmetric encoder-–decoder U-Blocks which are connected by M-Block through concatenation layers for feature fusion at different levels. Initially, we train our model by simulatively introducing five common biased colors. The various image quality indicators holistically demonstrate that our model achieves accurate color correction for tongue images, and simultaneously surpasses conventional algorithms and shallow networks. Furthermore, we conduct correction experiments by introducing random degrees of color bias, and the model continues to perform well for achieving excellent correction effects. The model achieves up to 84% correction effectiveness in terms of color distance ΔE for tongue images with varying degrees of random color cast. Finally, we obtain excellent color correction for actual captured images for tongue diagnosis application. Among these, the maximum ΔE can be reduced from 30.38 to 6.05. Overall, the TococoNet model possesses excellent color correction capabilities, which opens promising opportunities for clinical assistance and automatic diagnosis.

## 1. Introduction

Tongue diagnosis is a crucial aspect of traditional Chinese medicine (TCM) diagnosis and treatment [1,2]. It offers the advantage of being non-invasive and convenient, as it analyzes human cases through examining the tongue’s condition. Traditional tongue diagnosis mainly relies on visual description, which encounters different situations that can affect color and lead to subjectivity [3]. Therefore, the current development direction of tongue diagnosis in modernizing Chinese medicine involves the application of computer vision technology for tongue image feature recognition [4]. The tongue, rich in features, closely correlates with health status [5], with the tongue color directly indicating abnormalities. However, shooting environments and lighting conditions are often inconsistent. This inconsistency leads to color bias in captured tongue images, making them different from the actual tongue appearance. Even the same tongue may appear differently under various shooting conditions, negatively impacting diagnosis [6,7]. Consequently, color correction of captured tongue image data becomes an essential component of automated diagnostic technology.

Early studies introduced various algorithms for color correction in different environments, such as methods based on Kernel Partial Least Squares Regression (K-PLSR) correction methods [6], backpropagation (BP) neural networks [7], white balance [8], neural network mapping [9], and polynomial regression methods [10,11]. Li Zhuo et al. [6,7] successively proposed color correction models based on simulated annealing (SA)-–genetic algorithm (GA)-–back propagation (BP) and K-PLSR. The SA-GA-BP algorithm can achieve a higher convergence rate than the classical BP algorithm. Meanwhile, K-PLSR converts linear regression to nonlinear and overcomes the difficulty of small data size. Both algorithms are based on Munsell ColorChecker for model training. Then, they substitute biased color tongue images into the model for correction. However, it affects the generalization ability of the model, and neither of them provide much interpretation or analysis of the correction results. Finlayson et al. [8] assumed that after the successful correction of white points in a biased color image, the other colors would be corrected as well. They used least squares and linear transformations for fitting. However, this white balance color correction method is not suitable for tongue images with excessive white points. Wang et al. [9] proposed an optimized BP neural network based on Munsell ColorChecker, aiming to avoid color correction in a particular imaging system. They also compared polynomial regression, ridge regression, and support vector regression algorithms. Wang et al. [11] compared polynomial regression algorithms, artificial neural networks, and support vector machine algorithms. They concluded that polynomial regression algorithms are more suitable for color correction of the tongue, while artificial neural networks are the least effective.

Additionally, the perfect reflection algorithm and derived algorithms, including the gray world algorithm based on standard bias weighting, were proposed [12]. In their study, Finlayson et al. presented the drawbacks of linear color correction (LCC) and conventional polynomial color correction (PCC). Then, they proposed a new root polynomial color correction algorithm. This algorithm avoids the possibility of significant errors in some areas. It also uses the Macbeth color chart to compare color bias before and after correction. Shukeri and Chen et al. performed color correction of tongue images based on device-independent LAB color space in comparison with Munsell color charts [13,14]. However, conventional polynomial regression algorithms have limitations in tongue color correction. These limitations arise from the requirement to cross-reference with standard color blocks.

At present, the medical field widely adopts artificial intelligence technology [15]. This technology extracts information from tongue images to identify physical features imperceptible to the human eye. It also offers new possibilities for color correction. Recent advancements have introduced convolutional neural networks for the color correction of tongue images [16,17]. These networks achieve superior results in objective color correction under diverse lighting conditions and present innovative ideas in the field. Convolutional neural networks are inspired by visual perception and mimic the way the brain works. Convolutional operations are used instead of general matrix multiplication operations, and therefore are more advantageous in the field of image data processing [18]. However, their algorithm only builds five convolutional layers. It does not apply pooling and batch normalization (BN) layers, leaving much room for improvement in the final correction results.

Previous tongue color correction methods have limitations in terms of correction results and do not take into account multiple color bias situations. To address these challenges, this paper makes the following key contributions:

(a) Deep Learning Architecture for Color Correction: We propose TococoNet, a deep convolutional neural network tailored for the regression task of tongue image color correction. Though TococoNet draws inspiration from U-Net’s topological structure, it adopts a more refined design. Specifically, this design incorporates symmetric U-Blocks to realize multi-scale feature extraction, alongside a dedicated M-Block that facilitates effective multi-level feature fusion. Together, these components enable the model to comprehensively integrate both local color details and global color context.
(b) Simulation-to-Real Training Framework: We introduced a robust framework that trains the model on simulated color-biased images. The model’s subsequent successful application to real captured images demonstrates the efficacy and strong generalization capability of this approach.
(c) Comprehensive Experimental Validation: We conduct extensive experiments, including corrections for five common and random degrees of color bias, demonstrating that TococoNet significantly outperforms conventional algorithms and an existing shallow CNN (DCCN). Furthermore, we validate the model’s practical utility by achieving excellent correction results on real captured tongue images.

The remainder of the paper is organized as follows. Section 2 describes classical color correction algorithms. Section 3 outlines the preparation of our proposed model and experiments. Section 4 presents our experimental data and comparison results. The final section discusses and concludes the proposed color correction algorithm.

## 2. Related Works

In this section, we present three previous color correction algorithms and a shallow convolutional neural network model, along with their respective computational formulas. The conventional algorithms rely on fixed formulas for color correction, lacking generalization capabilities and yielding subpar correction results. While the shallow convolutional neural network demonstrates improved color correction performance, it falls short in quantitative analysis indices. To address the limitations of deeper networks in tongue color correction, we propose TococoNet, a deep convolutional neural network (CNN) model, which offers excellent generalization ability for color correction.

### 2.1. Gray World Algorithm

Xin et al. [19] proposed a color correction method rooted in the gray world principle. Initially, the dimensionality of the captured tongue image is reduced twice. This reduction decreases the data volume substantially to merely 3.6%. It also remarkably enhances the efficiency of subsequent processing. Following this reduction, a color bias detection is executed on the streamlined image. Any images exhibiting color bias are then subjected to color correction using the gray world method. Kwok et al. [20] also utilize the gray world algorithm for color correction. They distinguish themselves from others by additionally employing a gamma correction method. It fulfills the prerequisites of the gray world algorithm while preserving image intensity and circumventing color saturation.

The fundamental premise of the gray world assumption is that average gray values of the RGB channels in a colorful image will converge to the same gray value, denoted as K. The gray world algorithm accomplishes color correction by adjusting the mean value of three channels in the biased color picture. The calculation formula is as follows:

(1) $$\left\{\begin{array}{l} K=(R_{avg}+G_{avg}+B_{avg})/3\\ R_{new}=R\times \frac{K}{R_{avg}},G_{new}=G\times \frac{K}{G_{avg}},B_{new}=B\times \frac{K}{B_{avg}} \end{array}\right.$$

Here, (*R_avg_*, *G_avg_*, *B_avg_*) are the mean values of the three-color channels. Meanwhile, (*R*, *G*, *B*) represent the pixel value of the original image in each color channel, and (*R_new_*, *G_new_*, *B_new_*) are the adjusted color components of the pixel.

### 2.2. Perfect Reflection Algorithm

The perfect reflection algorithm, also known as the specular method, operates on the alternative assumption that a white object in an image has extremely large *R*, *G*, and *B* values under any light source. Therefore, this algorithm follows a clear principle. It assumes the brightest point in the image is white, then uses this white point as a reference for white balance to achieve color correction. The calculation steps are as follows:

(2) $$R_{new}=\frac{R_{w}}{R_{\max}}\times R,G_{new}=\frac{G_{w}}{G_{\max}}\times G,B_{new}=\frac{B_{w}}{B_{\max}}\times B$$

Here, (*R_max_*, *G_max_*, and *B_max_*) represent the color components of the pixel, which are the maximum value of (R + G + B) in the image. Here, (*R_w_*, *G_w_*, and *B_w_*) are generally taken as 255 each.

In references [12,21], Liu and Xu et al. proposed a color correction algorithm by combining the gray world and perfect reflection methods (SDW), leveraging the advantages of both methods. The algorithm first divides the image into 16 × 16 blocks and calculates the color component weighted mean values of the image. Assigning different weights based on the standard bias of the pixels in each block reduces the effect of large swaths of a single color on the channel mean and better corrects the image.

### 2.3. Polynomial Regression Algorithm

The correction principle of the polynomial regression algorithm is to calculate the polynomial matrix of the mapping between the off-color color blocks and the standard color blocks, and then substitute the matrix into the overall image to achieve image correction. The calculation process is as follows, where *X* is the 3-channel matrix of the surrogate and *V* is the polynomial regression matrix.

(3) $$V_{out}={\left({\left(V\times V^{T}\right)}^{-1}(V\times X^{T})\right)}^{T}\times V_{in}$$

Wang et al. [11] conducted a comparative analysis of polynomial regression algorithms, artificial neural networks, and support vector regression. They ultimately concluded that polynomial regression algorithms are more suitable for the color correction of tongue images. Sui et al. [22] implemented a root polynomial regression approach. They incorporated the pth root of the pth polynomial term, effectively eliminating nonlinear variations in certain regions caused by variations in lighting conditions.

Polynomial regression-based algorithms are typically coupled with standard color blocks to correct the biased color blocks to standard color blocks. In Section 4.2, we selected the ColorChecker standard color card. This color card is produced by Menzel Laboratories, a subsidiary of AISI. It has 24 color blocks, and each block represents the actual color of natural objects.

### 2.4. Convolutional Neural Networks

Lu et al. [16,17] introduced a color correction model named deep-color correction network (DCCN) based on a convolutional neural network. They constructed a regression model comprising just five convolutional layers. This model is segmented into three parts: the input layer, the nonlinear transformation layers, and the output layer. Their use of a 40 × 40 patch for training the model provides valuable insights. It is noteworthy that they provide a color adjustment feature. This feature leverages three supplementary parameters to enable precise manipulation of color correction. In turn, this capability accommodates a wide range of work environments.

## 3. Method Overview

We employ a convolutional neural network to execute color correction on tongue images. Initially, we introduce a novel deep model capable of handling various color bias situations. Subsequently, we simulate five distinct tongue color biases, including lower brightness, higher brightness, and overall reddish, bluish, and greenish colors. Furthermore, we conduct a thorough evaluation of the correction’s efficacy using diverse metrics and compare our approach with alternative methods.

### 3.1. Network Architecture

**Figure 1:**
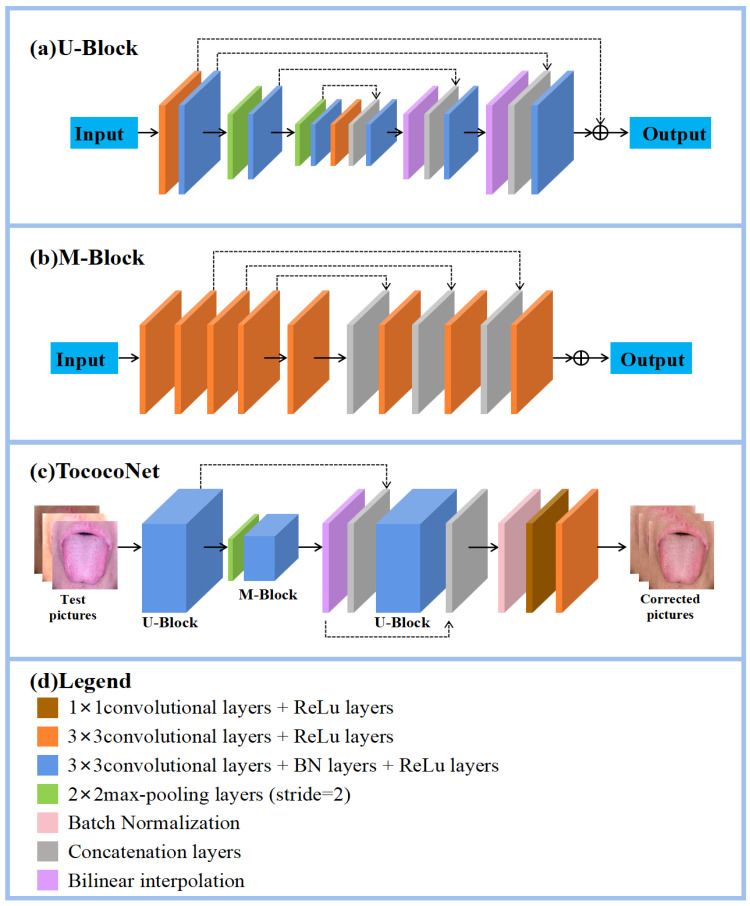
Overview of the TococoNet. (**a**) U-Block, tasked with encoder and decoder. (**b**) M-Block responsible for connecting the encoder and decoder. (**c**) Framework for the entire network. (**d**) Role of each layer.

U-Net, a semantic segmentation model based on convolutional neural networks as proposed in [23], is widely recognized for its efficient and accurate segmentation capability. In this work, we proposed a novel regression model for color correction inspired by the U-Net architecture. The TococoNet, combining downsampling coding and upsampling decoding, demonstrates excellent color correction abilities under various bias situations. The architecture of our model is shown in Figure 1.

The U-Block module is a symmetric encoder-–decoder structure, similar to that of U-Net. It is responsible for handling the encoding and decoding tasks. To better understand the image content, it gradually reduces the feature map size by stacking multiple convolutional and pooling layers. The encoder path employs successive convolutional and downsampling layers to capture multi-scale contextual information. The decoder path then upsamples the features to restore spatial resolution. A key feature of our U-Block is the use of internal residual connections, which help to propagate original color and texture information through the network, mitigating the information loss that can occur in very deep networks. Downsampling employs a 2 × 2 max pooling operation while upsampling uses bilinear interpolation, preserving smoothness and detail information. To prevent significant loss of contextual information, U-Block performs downsampling operations, adjusting the pooling step accordingly. Feature map size is restored to its original size after two upsampling passes. Feature map concatenation (concat) operations in each layer of downsampling and upsampling enhance the extraction of feature information, boosting the network model’s expressiveness.

The M-Block connects the encoder and decoder, achieving multi-scale and multi-level information fusion by linking bottom layer features with top layer features. Its core structure consists of two primary components: a block containing 8 convolutional layers, followed by a critical fusion operation. Maximum pooling and scales yield the final color-corrected image. The difference from U-Block is that the image scale remains unchanged during feature extraction with M-Block. The M-Block helps the model to better integrate features from different scales and levels, which is beneficial for color correction as it allows the model to consider both local and global color information.

The TococoNet model consists of two U-Blocks and one M-Block with the use of concatenation layers for fusion of features at different levels. The first U-Block uses 32 intermediate filters and outputs 64 channels. The M-Block uses 32 filters and outputs 64 channels. The second U-Block uses 32 intermediate filters and outputs 64 channels. The output path consists of a Batch Normalization (BN) layer, a 1 × 1 convolutional layer with 64 filters, and a final 3 × 3 convolutional layer with 3 filters. The architecture incorporates a total of 26 convolutional layers, with its depth providing the necessary capacity to learn the complex, non-linear mapping for color correction. A pivotal design choice is the strategic placement of Batch Normalization (BN) layers, particularly following the final feature fusion, which stabilizes training and acts as a regularizer for this deep regression network.

For offline training, we use MSEloss as the model’s loss function, calculated as:

(4) $$Loss(x)=\frac{1}{N}\sum_{i=1}^{N}{\left(F(x_{i})-L(x_{i})\right)}^{2}$$

where *F* and *L* represent the network output and real information, respectively, and *x* is the input position. The model was trained using the Adam optimization algorithm.

### 3.2. Training Environment

The training of this model was conducted on an Ubuntu 20.04 operating system using the PyTorch deep learning framework. The batch size was set to 32, and the model was trained for 200 epochs. We used the Adam optimizer with an initial learning rate of 1 × 10^−4^ and L2 weight decay of 1 × 10^−5^. A learning rate scheduler was employed, which reduced the learning rate by a factor of 0.5 if the validation loss plateaued for 15 epochs. Furthermore, we utilize three graphics cards for parallel training, thereby significantly boosting the training efficiency. The specific experimental environments, including the graphics card and CPU, are detailed in Table 1.

### 3.3. Evaluation Indicators

(a) ΔE: Chromatic distance ΔE, based on the CIE LAB color space, is an evaluation index calculated as:

(5) $$\Delta {E^{*}}_{ab}=\sqrt{{\left(\Delta L^{*}\right)}^{2}+{\left(\Delta a^{*}\right)}^{2}+{\left(\Delta b^{*}\right)}^{2}}$$

The LAB color space, composed of one luminance and two color channels, ensures uniform perception and device independence for human color sensation. Smaller ΔE indicates closer similarity to the original image. In this research, we use the ΔE for the initial detection of color bias. We derived the general criteria for ΔE from the results of our numerous experiments and the quantitative analysis of combined metrics and visual observations. The general criteria for ΔE are classified as follows: when ΔE < 6, it can be considered the same as the original image; when ΔE < 15, it is a mild color bias; when ΔE > 15, it is a color bias. In the evaluation, we categorize chromatic distance into five levels, namely the average color moment, the highest color moment, and the first (Q1), second (Q2) and third (Q3) quartiles, to comprehensively assess image quality.

(b) MAE and RMSE: Mean Absolute Error (MAE) and Root Mean Square Error (RMSE) measure the difference between predicted and actual values. They are, respectively, defined as:

(6) $$MAE=\frac{\sum_{i=1}^{n}\left|y_{i}-x_{i}\right|}{n}$$

(7) $$RMSE=\sqrt{\frac{\sum_{i=1}^{n}{\left(y_{i}-x_{i}\right)}^{2}}{n}}$$

Smaller values for both MAE and RMSE indicate a better fit of the model.

(c) PSNR: Peak Signal to Noise Ratio (PSNR) evaluates image quality, with larger values indicating less distortion. The PSNR is calculated using the Mean Squared Error (MSE):

(8) $$MSE=\frac{\sum_{i=1}^{n}{\left(y_{i}-x_{i}\right)}^{2}}{n}$$

(9) $$PSNR=10\times \log_{10}(\frac{MA{X_{I}}^{2}}{MSE})=20\times \log_{10}(\frac{MAX_{I}}{RMSE})$$

where *MAX_I_* represents the maximum pixel value of the image, usually set to 255.

(d) SSIM: The Structural Similarity Index Measure (SSIM) determines the similarity of two images, more aligned with human visual characteristics than PSNR. It ranges between 0 and 1, with a greater value indicating higher similarity, and the value 1 indicating identical images. The SSIM is computed as:

(10) $$SSIM(x,y)=\frac{\left(2\mu_{x}\mu_{y}+c_{1})(2\sigma_{xy}+c_{2}\right)}{\left({\mu_{x}}^{2}+{\mu_{y}}^{2}+c_{1})({\sigma_{x}}^{2}+{\sigma_{y}}^{2}+c_{2}\right)}$$

where *μ_x_* and *μ_y_* represent the mean, *σ_x_* and *σ_y_* represent the variance, *σ_xy_* represents the covariance, and *c_1_* and *c_2_* are constants used to maintain stability.

### 3.4. Training Dataset

**Figure 2:**
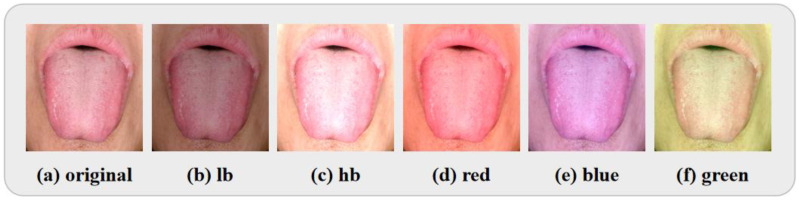
Datasets created by simulating bias color about five common off-colors, from left to right, are (**a**) origin image, (**b**) lower brightness image, (**c**) higher brightness image, (**d**) overall reddish image, (**e**) overall bluish image, and (**f**) overall greenish image, respectively.

For the acquisition of tongue images, we employed an instrument to simulate natural light and ensure uniform illumination. The color temperature of the instrument was set to 6600 K, the illuminance was around 3000, and the color rendering index was 90%. We collected a total of 577 tongue images and finally selected 507 of them for training and testing after quality screening. The acquired tongue images were processed for biased color, resulting in the creation of a dataset in different states. We simulated five common bias color conditions, as illustrated in Figure 2: lower brightness (lb), higher brightness (hb), and overall reddish, bluish, and greenish colors. The simulation formulas for lower and higher brightness are as follows:

(11) color=color∗contrast±brightness

where the *color* refers to the adjusted brightness, the *contrast* takes values in the range of 0.8–1, and the *brightness* refers to the offset that takes values in the range of 0–30.

The simulation formula for overall reddish, bluish and greenish is as follows:

(12) color=min(255,color+adjustment)

where the *color* refers to each color component and *adjustment* is taken as 50. Due to the numerical limitations of the RGB format, each channel will be clipped after adjustment. If the adjusted color value exceeds 255, it will be set to 255. The brightness adjustments are not subject to this limitation, so no additional cropping was performed. After multiple adjustments to these parameters, the simulated biased color images are similar to the actual biased color.

Insufficiency of the hardware conditions in need of training with the whole image requires us to use color blocks. The images were uniformly sized at 640 × 640 and cropped into 160 × 160 color blocks with a step of 80 to construct the training, validation and test dataset. The ratio of the training set, validation set, and test set is 60%, 30%, and 10%.

## 4. Results

In this section, we present three distinct experimental outcomes pertaining to the proposed model. Firstly, we showcase the effectiveness of the TococoNet in correcting biased color blocks. Secondly, we conduct a comparative analysis with alternative algorithms. Finally, to further scrutinize the model’s correction capabilities, we present the color correction results for randomly biased tongue images.

### 4.1. Correction Results of Color Blocks

**Figure 3:**
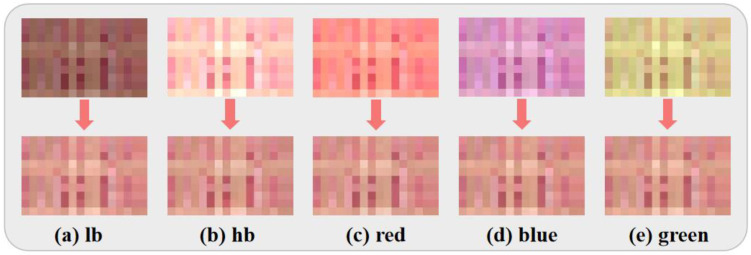
Correction results for color blocks, from left to right, are (**a**) lower brightness image, (**b**) higher brightness image, (**c**) overall reddish image, (**d**) overall bluish image, and (**e**) overall greenish image, respectively.

First, since the TococoNet model is trained on a dataset built around color blocks, we create new color blocks by extracting individual pixels from the tongue images after simulating bias color. The model is then used to correct these color blocks under different bias conditions. The biased color blocks and the correction results are depicted in Figure 3. According to qualitative observations, a pixel-level comparison reveals the model’s excellent color correction capabilities. It is evident that there exists a substantial disparity in color among the color blocks prior to correction. Following the application of the model’s correction, the colors of these blocks become strikingly similar, demonstrating the effective correction of the five bias situations.

**Figure 4:**
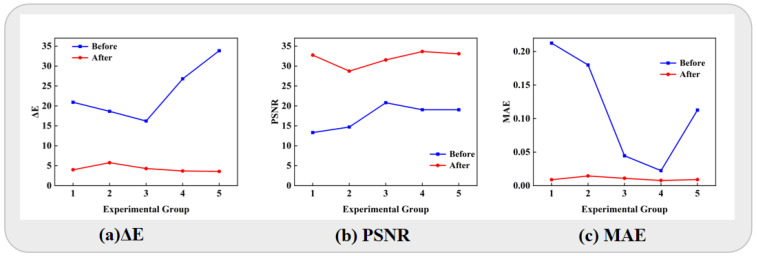
Evaluation curves of correction results for pixel color blocks. (**a**) Chromatic distance (ΔE). (**b**) Peak Signal to Noise Ratio (PSNR). (**c**) Mean Absolute Error (MAE). Evaluation indicators of the corrected images are significantly improved.

We choose ΔE, PSNR, and MAE for the initial evaluation of model performance with respect to color block correction. As illustrated in Figure 4, the ΔE values for the five biased color cases are reduced compared to before correction, and the PSNR values also show improvement. The MAE value is also decreased, indicating a reduction in the error between the corrected and the original images. Specifically, as depicted in Figure 4a, the ΔE difference before and after correction is minimal for a reddish situation, whereas it is most significant for a greenish situation. This is attributed to the fact that most tongue colors exhibit red, while greenish hues are quite uncommon. The PSNR curves in Figure 4b for the five color-biased scenarios show relatively flat trends. This suggests that corrected images across different color biases have similar graphic quality. As seen in Figure 4c, the case of a bluish situation yields the smallest pixel difference between the biased and standard images, while lower brightness cases result in the largest disparity.

**Figure 5:**
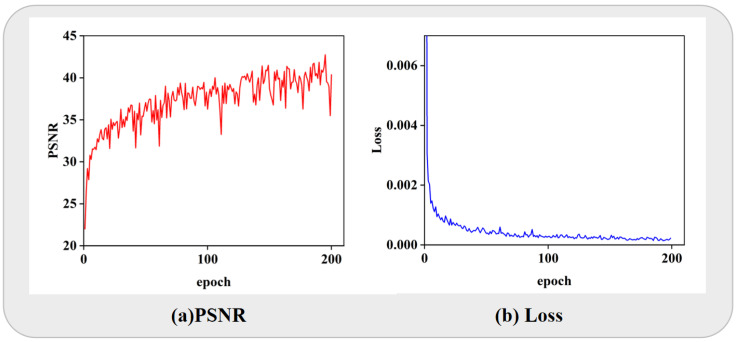
Training results for color blocks. (**a**) PSNR. (**b**) Loss function.

Furthermore, we conduct a thorough analysis of the evolution of the PSNR value and the loss function during the training of the TococoNet. We plotted the corresponding curves in Figure 5 to visually illustrate these changes. As the training progressed, the PSNR value of the training set gradually ascended, indicating an improvement in the model’s performance. It reached a peak value of 43 ultimately, suggesting a significant enhancement in image quality. The curves of the loss function also revealed that the training process was relatively stable, exhibiting gradual convergence and avoiding any signs of overfitting.

### 4.2. Comparison with Other Algorithms

**Figure 6:**
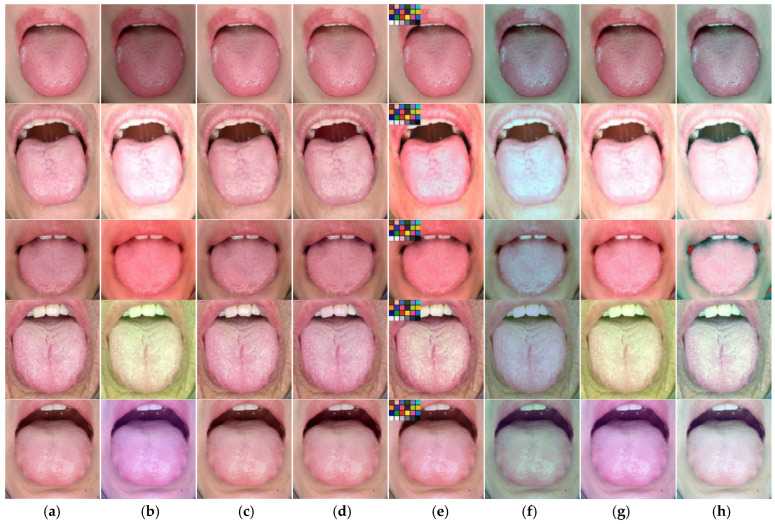
Correction results using the TococoNet and other algorithms. (**a**) Original tongue images. (**b**) Biased color images, (**c**) TococoNet, (**d**) DCCN, (**e**) Polynomial regression algorithm, (**f**) Gray world algorithm, (**g**) Perfect reflection algorithm, (**h**) SDW algorithm. TococoNet and DCCN corrections are significantly more excellent than the other algorithms.

To further verify the validity of our model, we applied color correction to the images in the dataset using the TococoNet and other algorithms. The correction results are presented in Figure 6. The first two columns from left to right are the original and biased color images. The remaining columns, from left to right, are images of the tongue after color correction using different methods. As shown in Figure 6e, we compared the results of different algorithms. The polynomial regression algorithm introduces a contrasting color block, which is due to its correction characteristics. Among the four conventional methods, the polynomial regression algorithm yields relatively good correction results, while the other algorithms fail to meet the standard requirements for color correction. It is evident that classical methods prove less effective in correcting biased color pictures of the tongue and are unsuitable for tongue color correction.

The outcomes of both CNNs models clearly demonstrate excellent color correction ability, yielding convincing results for various cases of color bias. As depicted in Figure 6c,d, the color correction results of the TococoNet and DCCN exhibit remarkable outcomes when compared to the biased color tongue images. The distinction between the corrected tongue image and the original is imperceptible to the naked eye, necessitating a further quantitative evaluation of the models’ correction efficacy.

To further emphasize the model’s correction ability, evaluation parameters for the calibration of the TococoNet and the DCCN are provided in Table 2, offering robust evidence of the model’s correction capability. After correction, the SSIM indicators of the TococoNet model surpassed 0.99, signifying the exceptional quality of the corrected tongue images. The average ΔE dropped below 2, while the maximum ΔE hovered around 20, demonstrating that the corrected images closely mimic the original ones. Furthermore, the PSNR witnessed a significant improvement, nearing 50, significantly enhancing the color quality of the tongue image. Additionally, the MAE and RMSR values underwent substantial reductions, confirming the minimization of differences between the corrected and original images.

However, upon examining the evaluation metrics, it becomes evident that the correction performance of DCCN lags behind that of the TococoNet. The PSNR value of DCCN’s correction results barely surpasses 30, and its SSIM value falls below 0.99, significantly trailing behind the TococoNet. Furthermore, the MAE and RMSE values of DCCN are significantly higher than those of the TococoNet, reflecting the inferior quality of its calibration. The detailed processing of the shallow network is inferior to our network, with the maximum ΔE even exceeding 100, which suggests a failure in correcting the individual pixel points. This is likely due to the model’s limited capacity and lack of batch normalization, which can lead to unstable training and poor generalization on outlier pixels. Almost all indicators of DCCN are a bit insufficient compared to the TococoNet.

To ensure the statistical robustness of our results and to quantify the variability of the model’s performance, we employed a bootstrap resampling approach. From the test set, we generated 40 bootstrap samples (with replacement) and evaluated the model on each. All reported performance metrics are subsequently presented as the mean ± standard deviation across these 40 runs. As shown in Table 3, the PSNR and ABC metrics of TococoNet were both superior to those of DCCN. And the significance of the performance differences between TococoNet and the DCCN was assessed using paired t-tests for each color bias scenario and metric. The significance of the performance differences between TococoNet and the DCCN baseline was assessed using paired t-tests for each color bias scenario and metric. A result was considered statistically significant at *p* < 0.001. As shown in Table 4, the performance difference between the two methods was found to be highly statistically significant.

### 4.3. Correction Results of Random Biased Color Images

**Figure 7:**
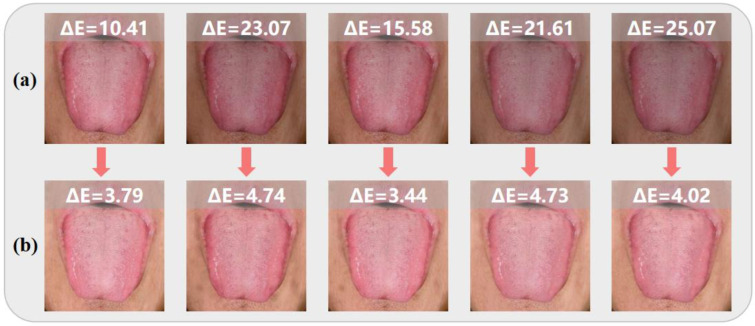
(**a**) Original tongue images. (**b**) Corrected images. Focused on lower brightness biased images, a significant reduction in the ΔE indicator indicates excellent correction results for random degrees of color bias.

To verify the stability and generalization ability of the model, we constructed a second dataset with a random degree of color bias using lower brightness biased images as an example. The element of uncertainty introduced by the random bias posed a greater challenge to the model’s generalization capabilities. Testing the trained model with randomly biased color images, as illustrated in Figure 7, reveals the model’s capability of correct color correction for different degrees of bias. After correction, the details of color in the tongue images were preserved, and tongues with varying degrees of bias color exhibited similar color performance. Evaluation using ΔE is labeled at the top of each picture, clearly indicating that our correction results have a lower ΔE compared to the off-color images. Specifically, among the five tongue images with ΔE ranging from 10.41 to 25.07, their ΔE values were all reduced to around 4 after color correction, with the most significant improvement achieving an 84% reduction.

### 4.4. Correction Results of Actual Biased Color Images

**Figure 8:**
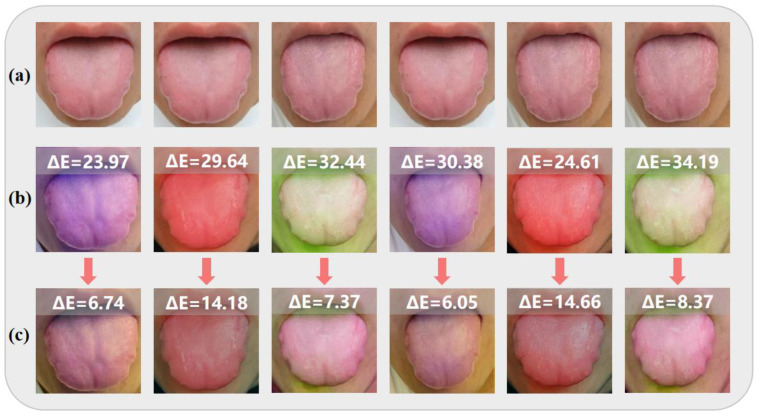
The correction effect of the biased color images taken in real life can have the same excellent correction effect. (**a**) Standard color images. (**b**) Biased images. (**c**) Corrected images.

To evaluate the correction effect of the trained model on real images, we captured color-biased tongue images under different colored lighting conditions and compared them with images obtained in a non-color-biased lighting environment. Since lower and higher brightness conditions are unfavorable for images, only color-biased scenarios were selected. For this initial investigation, we captured images from two different tongues under three distinct colored light sources (red, green, blue). This resulted in a total of 6 real color-biased tongue images for testing. As shown in Figure 8, (a) represents the images under non-color-biased lighting, (b) shows the color-biased images, and (c) displays the corrected images. We continued to use ΔE as the evaluation metric, which is annotated above each image. The correction outcomes were favorable for images biased towards blue and green colors, while overall reddish images exhibited slightly less effective correction. Among them, the ΔE of the overall bluish tongue images can be reduced from 23.97 and 30.38 to around 6, while that of the images with overall greenish colors can drop from 32.44 and 34.19 to approximately 7 and 8, respectively.

**Figure 9:**
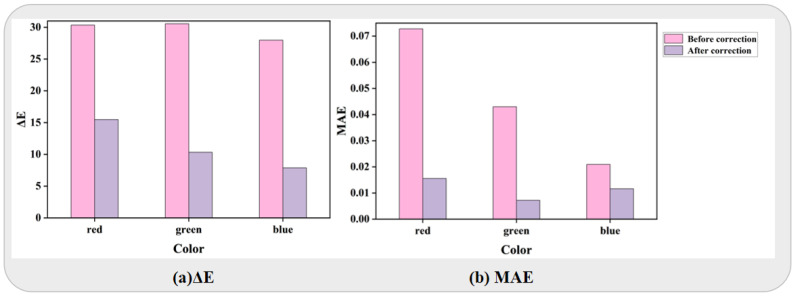
Comparative results of real-world color correction testing. (**a**) Chromatic distance (ΔE). (**b**) Mean Absolute Error (MAE).

More comprehensive and extensive results of the real-world experiments are shown in Figure 9. We visualized the correction results utilizing both ΔE and MAE metrics. Notably, the correction of overall bluish tongue images exhibits superior performance based on the ΔE metric, whereas the reddish color correction falls behind. Conversely, according to the MAE metric, the reddish correction yields the highest effect, with the bluish correction demonstrating the opposite trend.

However, for images with reddish colors, the ΔE can only be lowered to about 14, indicating significant room for further improvement. The reason why reddish images showed less effective correction is that the color of tongue itself is red. And the tongue color is similarly difficult to label when categorizing red features. Specific red, deep red, pale red and other colors are difficult to distinguish when they are close to each other [15]. In tongue diagnosis, different shades of red, such as pale red, bright red, and crimson red, correspond to different physiological states. During training, the model may receive contradictory signals: for an image with a reddish tint, the red color in some areas is a “bias” that needs correction, while the red color in other areas is a “feature” that needs retention. This ambiguity at the semantic level makes the model’s learning objective unclear, causing it to tend toward conservative processing and thus resulting in insufficient correction.

## 5. Discussion

The experimental results in Section 4 show that our proposed model can effectively correct tongue images after bias coloring. In comparison to other methods, our utilization of a deep convolutional neural network for regression exhibits superior performance. The advantage of our model lies in its deeper architecture compared to Lu et al. [16]’s model. It not only enhances its performance but also effectively avoids the issue of overfitting. This approach is exemplified by Steffens et al. [24], who employed a deep network to conduct correction experiments on images with inadequate exposure.

However, it is noteworthy that this study also possesses certain limitations and holds the potential for further improvement. Firstly, owing to the unique nature of the deep learning approach, our experiments are simulated bias color experiments that are conducted within a controlled laboratory setting. While the experimental data unequivocally demonstrates the model’s effective color correction capabilities, there are numerous challenges that may arise when applying the model for correction in real-world scenarios. Secondly, the model demonstrates relatively inferior performance in correcting reddish color casts compared to blue and green ones, as evidenced by the higher residual ΔE in real-image experiments. Quantitative and more in-depth research will be conducted to analyze the causes in the future. Furthermore, our network lacks optimization mechanisms and holds significant potential for further enhancement and improvement.

As part of our future research, we intend to initially fine-tune the network’s details and introduce structure optimization mechanisms. Optimizing the processes of training and prediction is also paramount, encompassing the improvement of the loss function and hyperparameters, as well as minimizing the training cost. We will explore perceptual loss functions, such as combining MS-SSIM (Multi-Scale Structural Similarity Index Measure) with L1 loss, with the aim of better preserving texture structures during the color conversion process. Secondly, we plan to experiment with other neural network architectures for designing a color correction model, and select the most suitable one through comparison for further optimization. Additionally, we aim to simulate various other biased color situations to enhance the model’s generalization capability and robustness. We will continue to conduct authentic bias color experiments to account for the influence of practical factors on tongue images. On the flip side, there is a notable scarcity of research concerning the detection of biased colors, which necessitates the urgent exploration of innovative ideas in this area. As part of our endeavor, we will design a lightweight color cast detection network to automatically identify the type and degree of color cast before correction. This pursuit is of equal importance in advancing the digitization of tongue diagnosis.

## 6. Conclusions

The widespread application of deep learning has opened new avenues for research directions in the field of tongue diagnosis. In response to the challenge of color correction in tongue images, a deep regression model named TococoNet is proposed to eliminate the color bias in these images. Initially, we simulate five common cases of color bias to train the model. The correction results of TococoNet are significantly effective, lifting the PSNR of tongue images to 40 and shrinking ΔE to 2. The accurate color correction results demonstrate a higher performance than conventional algorithms and shallow networks. Furthermore, we conduct correction experiments for random degrees of color bias, achieving an excellent correction effect as well. Finally, we also achieve excellent results when applying the correction to actual captured images. Among these, the correction effect is better for the bluish and greenish tongue images, while the effect on the reddish images is relatively average. In summary, the TococoNet model possesses excellent color correction capabilities, which can offer promising opportunities for clinical support and automatic diagnosis.

## Figures and Tables

**Table 1:** Experimental environment.

Entry	Version
GPU	Nvidia RTX4090
CPU	Intel Xeon Silver 4316
CUDA	11.6
Python	3.7
Numpy	1.26.4
Opencv	4.6.0.66
System	Ubuntu 20.04
PyTorch	1.12.0

**Table 2:** Evaluation indicators for the correction results of our model and the DCCN model. The first of these columns shows the five cases of biased color. The second column presents the different correction states, which are the initial tongue image before correction, after being corrected by our model, and DCCN, respectively. The remaining columns are different evaluation indicators.

Color Bias	Correction State	PSNR	SSIM	E_mean	E_Q1	E_Q2	E_Q3	E_Max	MAE	RMSE
lb	Original	13.7290	0.9100	20.2729	19.7567	20.5144	21.0200	25.1379	0.2019	0.2031
	Ours	**43.2377**	**0.9940**	**1.3028**	**1.0000**	**1.2709**	**1.6171**	**17.9678**	**0.0050**	**0.0063**
	DCCN	36.3999	0.9762	2.5581	1.2709	2.0000	3.0000	118.3315	0.0058	0.0103
hb	Original	14.8771	0.9532	19.0591	17.7407	18.5665	20.6949	29.3753	0.1753	0.1768
	Ours	**36.4937**	**0.9907**	**2.4330**	**1.2709**	**2.0381**	**3.1623**	**17.0541**	**0.0059**	**0.0084**
	DCCN	33.1935	0.9815	4.3246	2.2706	3.7926	6.0510	129.9648	0.0095	0.01296
red	Original	19.1908	0.9871	21.6838	21.6436	22.3689	23.0614	33.1298	0.0566	0.0570
	Ours	**40.9000**	**0.9955**	**1.4789**	**1.0741**	**1.2709**	**1.6171**	**11.2078**	**0.0061**	**0.0071**
	DCCN	34.0676	0.9622	4.0921	2.3696	3.6268	5.1590	106.6675	0.0112	0.0142
green	Original	18.9848	0.9807	34.0577	33.6523	34.1437	34.9353	44.6470	0.1136	0.1139
	Ours	**43.5519**	**0.9957**	**1.3325**	**1.0000**	**1.2709**	**1.4676**	**20.6038**	**0.0041**	**0.0061**
	DCCN	32.2688	0.9711	4.7157	3.4413	4.4547	5.4630	88.9739	0.0206	0.0237
blue	Original	18.9457	0.9973	27.6570	26.9515	27.2481	27.9175	35.5181	0.0227	0.0228
	Ours	**43.2933**	**0.9961**	**1.6585**	**1.0741**	**1.4676**	**2.2361**	**17.9950**	**0.0028**	**0.0040**
	DCCN	33.1320	0.9667	3.2881	1.4676	2.2361	3.6268	128.5556	0.0124	0.0207

**Table 3:** Evaluation indicators for the bootstrap resampling results between our model and the DCCN model. For key metric (PSNR, ΔE), we report the mean ± standard deviation.

Indicator	Model	lb	hb	Red	Green	Blue
PSNR	Ours	43.31 ± 1.53	35.31 ± 1.53	39.85 ± 1.23	43.31 ± 1.53	42.80 ± 1.96
	DCCN	36.4 ± 1.48	32.92 ± 1.19	34.44 ± 0.89	31.92 ± 1.19	34.85 ± 2.60
ΔE	Ours	1.69 ± 1.46	2.60 ± 1.40	1.69 ± 1.46	1.78 ± 1.45	1.67 ± 1.47
	DCCN	3.22 ± 1.44	4.72 ± 1.44	4.22 ± 1.42	4.36 ± 1.38	3.57 ± 1.33

**Table 4:** Evaluation indicators for the paired t-tests comparing the bootstrap distributions of TococoNet against the DCCN. The t-value and *p*-value are used to evaluate the statistical significance of the performance difference (*p* < 0.001 indicates an extremely significant difference).

Indicator	t/*p*	lb	hb	Red	Green	Blue
PSNR	t	18.65	7.11	20.63	33.93	15.19
	*p*	4.93 × 10^−21^	1.54 × 10^−8^	1.37 × 10^−22^	1.45 × 10^−30^	5.69 × 10^−18^
ΔE	t	−13.77	−20.48	−22.78	−31.90	−24.42
	*p*	1.46 × 10^−16^	1.79 × 10^−22^	3.86 × 10^−24^	1.47 × 10^−29^	3.06 × 10^−25^

## Data Availability

The datasets used and analyzed during the current study are available from the corresponding author upon request.
